# Concomitant influence of helminth infection and landscape on the distribution of Puumala hantavirus in its reservoir, *Myodes glareolus*

**DOI:** 10.1186/1471-2180-11-30

**Published:** 2011-02-08

**Authors:** Alexis Ribas Salvador, Emmanuel Guivier, Anne Xuéreb, Yannick Chaval, Patrice Cadet, Marie-Lazarine Poulle, Tarja Sironen, Liina Voutilainen, Heikki Henttonen, Jean-François Cosson, Nathalie Charbonnel

**Affiliations:** 1 Laboratori de Parasitologia, Departament de Microbiologia i Parasitologia Sanitaries, Facultat de Farmacia, Universitat de Barcelona, Barcelona, Spain; 2 INRA, UMR CBGP (INRA/IRD/Cirad/Montpellier SupAgro), Campus international de Baillarguet, CS 30016, F-34988 Montferrier-sur-Lez cedex, France; 3 2C2A-CERFE, 5 rue de la Héronnière, 08240 Boult-aux-Bois, France and Université de Reims Champagne Ardenne, Laboratoire de Parasitologie, EA 3800, 51092 Reims Cedex, France; 4 Infection Biology Research Program, Haartman institute, Department of Virology, PL 21, FI-00014 University of Helsinki, Helsinki, Finland; 5 Finnish Forest Research Institute, PL 18, FI-01301, Vantaa, Finland

## Abstract

**Background:**

Puumala virus, the agent of nephropathia epidemica (NE), is the most prevalent hantavirus in Europe. The risk for human infection seems to be strongly correlated with the prevalence of Puumala virus (PUUV) in populations of its reservoir host species, the bank vole *Myodes glareolus*. In humans, the infection risks of major viral diseases are affected by the presence of helminth infections. We therefore proposed to analyse the influence of both helminth community and landscape on the prevalence of PUUV among bank vole populations in the Ardennes, a PUUV endemic area in France.

**Results:**

Among the 313 voles analysed, 37 had anti-PUUV antibodies. Twelve gastro-intestinal helminth species were recorded among all voles sampled. We showed that PUUV seroprevalence strongly increased with age or sexual maturity, especially in the northern forests (massif des Ardennes). The helminth community structure significantly differed between this part and the woods or hedgerows of the southern cretes pre-ardennaises. Using PUUV RNA quantification, we identified significant coinfections between PUUV and gastro-intestinal helminths in the northern forests only. More specifically, PUUV infection was positively associated with the presence of *Heligmosomum mixtum*, and in a lesser extent, *Aonchotheca muris-sylvatici*. The viral load of PUUV infected individuals tended to be higher in voles coinfected with *H. mixtum*. It was significantly lower in voles coinfected with *A. muris-sylvatici*, reflecting the influence of age on these latter infections.

**Conclusions:**

This is the first study to emphasize hantavirus - helminth coinfections in natural populations. It also highlights the importance to consider landscape when searching for such associations. We have shown that landscape characteristics strongly influence helminth community structure as well as PUUV distribution. False associations might therefore be evidenced if geographic patterns of helminths or PUUV repartition are not previously identified. Moreover, our work revealed that interactions between helminths and landscape enhance/deplete the occurrence of coinfections between PUUV and *H. mixtum *or *A. muris-sylvatici. *Further experimental analyses and long-term individual surveys are now required to confirm these correlative results, and to ascertain the causal links between helminth and PUUV infection risks.

## Background

Puumala virus (PUUV) is the most prevalent hantavirus in Europe [1,2]. It is the agent of a mild form of hemorrhagic fever with renal syndrome called nephropathia epidemica (NE). The main course of transmission to humans is indirect by inhalation of virus-contaminated aerosols [3] from excreta of infected bank voles, *Myodes glareolus*, the reservoir of PUUV [4,5]. In France, about 60 cases of NE are yearly notified, but up to 250 cases can be observed during epidemic years (Data from the Institut National de Veille Sanitaire, INVS). The most important endemic areas of NE, which account for 30-40% of the human cases, are located in the Ardennes, along the Belgian border [6,7].

The risk for human infection seems to be strongly correlated with *M. glareolus *population abundance [e.g. [8]], which shows multi-annual fluctuations driven in temperate Europe by variations in tree seed production [9,10]. It is also related to the spatial distribution of PUUV-infected rodents, which depends on diverse factors including rodent community structure [11-14] or landscape features [15-17]. Patch size, fragmentation and isolation of landscape may influence the dispersal of voles and consequently the epidemiology of PUUV [15]. In addition, different characteristics of the soil such as moisture may affect the survival of PUUV in the natural environment, therefore influencing the importance of an indirect transmission of this hantavirus among rodents [18,19].

Landscape features are also strong determinants of the macroparasite community structure [20]. Interestingly, recent reviews have stressed the importance of helminth coinfection for viral disease epidemiology [21,22]. Such infections could lead to variations in the outcome of virus infection through direct or indirect mechanisms. First, helminths and viruses might compete either for food or space. For example, helminths that induce anemia could limit the replication of viruses that depend on red blood cells [see, [21]]. Second, host immunity may modulate the outcomes of helminth-virus coinfection through immunosuppression or cross-immunity [21-23]. In the majority of cases, helminth infections induce a polarisation of the immune response to Th2, and a down-regulation of the Th1 cell-subset [24,25]. They may also induce immunomodulatory mechanisms [24]. As such, the risks of infections and the severity of major viral diseases of humans (e.g. HIV, Hepatitis B and C) are known to be affected by the presence of many helminthic infections [e.g. *Schistosoma mansoni, Ascaris*, see [26-28]].

To our knowledge, there is no study that investigated this question of the potential concomitant influence of helminth community structure and landscape on the risk of hantavirus infection, either in humans, laboratory animals or natural reservoir populations. We explored this issue by analysing the interspecific interactions between gastro-intestinal helminths and PUUV among a cross-sectional natural population sample of bank voles trapped in different landscapes of the Ardennes, the main PUUV endemic area in France.

## Methods

### Bank vole sampling and parasitological screenings

**Figure 1:**
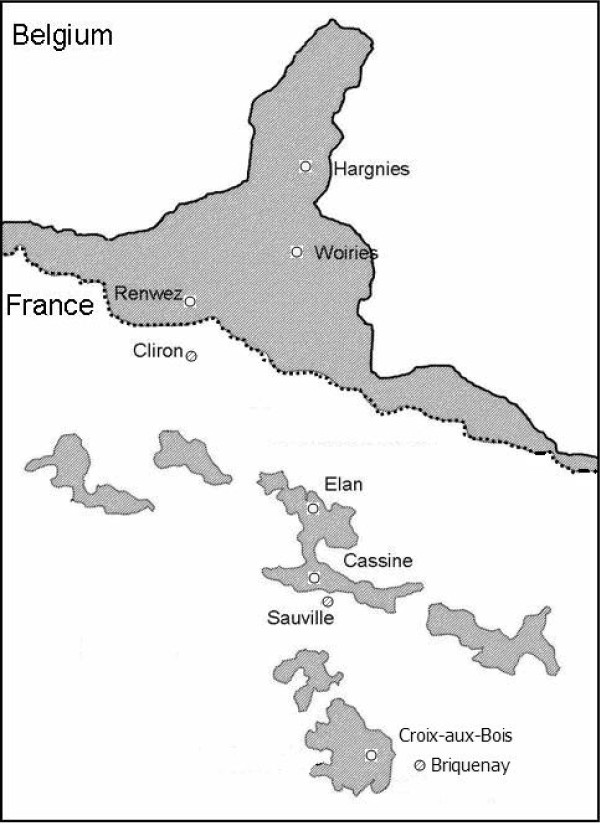
**Sampling localities for *M. glareolus *in the French Ardennes**. Forests and wooded areas are indicated in grey. White circles correspond to forested areas of the Northern massif des Ardennes. White and dashed circles respectively correspond to wooded areas and hedge networks of the Southern crêtes pré-ardennaises. The dashed line indicates the limit between the Northern massif des Ardennes and the Southern crêtes pré-ardennaises. Numbers refer to site codes indicated in Table 1.

Bank voles were sampled from September to October 2008 as PUUV and helminth prevalence levels are usually higher in autumn, which corresponds to the end of the reproductive season [e.g. among many studies [29,30]]. We used French Agricultural Research Institute (INRA) live traps, fitted out with dormitory boxes and baited with potatoes and sunflower seeds. Nine sampling sites were surveyed along a North - South transect in the French Ardennes. They corresponded to three different landscape configurations: forests, which are found in the northern 'massif des Ardennes' and refer to large wooded areas of several thousand hectares, smaller forest fragments (wooded areas of about 50 km^2^) and hedge networks surrounding these fragments, which are found in the Southern 'crêtes pré-ardennaises' (Figure 1). Ten 200-m trap-lines composed of 20 traps placed at 10-m intervals were placed within each site. They were checked twice a day during three consecutive nights. The minimum distance between sites was 3.2 km, that is much larger than the dispersal distance of bank voles [estimated to be 500 m in patchy landscapes, [31]].

Once trapped, voles were sacrificed by cervical dislocation as recommended by Mills et al. [32]. They were sexed and weighted. Body length was measured from snout to vent to the nearest 1 mm. Body condition of bank voles was estimated as the body mass index [BMI = weight/length2, [33]]. Animals were dissected. The sexual maturation of bank voles was deduced from testes and uterus size by visual observation. Males with developed epididymis were considered as sexually mature. Females with uterus smaller than 1 mm were considered as nullipare. We also distinguished females that were in gestation or lactation (uterus larger than 3 mm, presence of fetuses or lactating mammary glands) from females that had previously reproduced (uterus size of 2 mm or uterine scars) but that were not reproducing at the time of sampling. The digestive tracts were removed and stored in 96% ethanol before being analysed in the laboratory. All the helminths detected were carefully counted under the microscope and identified unambiguously using morphological criteria. For each individual, blood samples were also taken from the heart or the thoracic cavity on a 1-cm^2 ^Whatman blotting paper.

All listed animal procedures were pre-approved by the Direction des Services Vétérinaires of the Herault Department (B 34-169-1 Agreement).

### PUUV serological screening and viral load quantification

In the laboratory, each piece of Whatman blotting paper was placed in 1 ml phosphate-buffered saline. These diluted blood samples were screened for IgG antibodies to Puumala virus (PUUV) using immunofluorescence antibody test (IFAT) as described in Lundkvist et al. [34].

PUUV load was measured in PUUV seropositive voles using real-time quantitative RT-PCR. Total RNA was extracted from lung tissue samples as PUUV concentration is high compared to other organs [35]. We used TriPure Isolation Reagent (Roche) according to the manufacturer's instructions. One μg of RNA was used for first-strand cDNA synthesis using RevertAid™ H Minus Kit (Fermentas) with random hexamers. Real-time quantitative PCR was done using a DyNAmo Capillary SYBR Green Quantitative PCR kit (Finnzymes) with a LightCycler instrument (Roche). The following primers (Oligomer) were used: PUUV-forward 5'-GAG GAT ATA ACC CGC CAT GA-3', PUUV-reverse 5'-CTG GCT TGC AGT GTG TTT TT-3'. Samples were first normalized against variation in vole lung sample quality and quantity to GAPDH expression with the following primers: GAPDH-forward 5'-ATG GGG AAG GTG AAG GTC G-3' and GAPDH-reverse 5'-TAA AAG CAG CCC TGG TGA CC-3'. We then provide an absolute quantification for PUUV RNA: PUUV copy numbers (copies per 1 μg of total RNA) were calculated from a standard curve created using 10-fold dilutions of in vitro transcribed PUUV S segment RNA (T7 transcription kit, Fermentas). Melting curve analysis was performed according to recommendations of the DyNAmo kit to confirm the specificity of positive samples. Samples were considered PUUV RNA positive when the *C*_*T*_(cycle threshold) value was lower than 40 cycles and the melting curve showed a specific product.

### Statistical analyses

A logistic regression was first applied to determine vole individual characteristics that best explained PUUV infection. The dependent variable was the presence/absence of anti-PUUV antibodies in voles. Sex, sexual maturity, mass, body condition, landscape and site nested within landscape were included as independent variables. All possible two way interactions were considered. Model selection was performed using the Akaike's Information Criterion [AIC, [36,37]]. The model with the lowest AIC value was viewed as the most parsimonious one, i.e. the one explaining most of the variance with the fewest parameters [36]. Nested models with difference of AIC <2 compared to the model with the lowest AIC were selected. Significance of explanatory factors and their interaction were determined using deletion testing, with the significance of a term determined by the log-likelihood ratio-test [38]. If the interaction term was significant, both lower order terms involved in that interaction were retained [39]. The sum of squares was used to test model fit (*F*-statistic). In *a posteriori *pairwise comparisons for least square means, a multiple comparison adjustment for the *p*-values were done according to the Tukey-Kramer method. These analyses were performed in Genstat 7.1 (Lawes Agricultural Trust, Rothamstead).

The helminth community structure was next analysed with regard to geographic parameters (site and landscape configuration). The helminth infracommunity structure was assessed by the number of helminth species. The prevalence (*i.e. *the proportion of voles infected) of each helminth species was estimated per site. Spatial variations of helminth co-occurrence/antagonism were explored using a correspondence analysis (CA) performed in ADE4 [40] and based on the presence/absence data of each helminth species per vole. Results were projected on the site map to illustrate geographic heterogeneity in helminth structure. Site/landscape differences along the two first CA axes were tested using non-parametric Kruskal-Wallis tests performed in Genstat 7.1 (Lawes Agricultural Trust, Rothamstead). We could therefore identify sites/landscape configurations exhibiting homogeneous helminth communities.

We used this partition to identify synergistic or antagonistic interactions between helminth species and PUUV infection. As such we avoided associations that would only be mediated by differences of helminth and PUUV distribution among landscapes. We applied the discriminant analysis (DA) performed in ADE4 [40] to maximize the variance between designated groups (PUUV seronegative vs seropositive voles) while keeping the intra-group variance constant [41]. The significance of the ratio of these two values was tested using 10,000 permutations. For each helminth, we estimated the relative risk following Haldane [42] and we tested the association with PUUV-serological status using Fisher exact tests followed by Bonferroni sequential corrections.

Finally, we considered PUUV infected voles to compare the viral load of individuals coinfected with helminths significantly associated with PUUV and individuals non-infected with these helminths. Under the assumption of a positive interaction between PUUV and a given helminth, we expected that PUUV viral load should be comparatively lower in PUUV-helminth coinfected voles than in voles only infected by PUUV [43].

## Results

### Helminth and PUUV data

A total amount of 313 bank voles was sampled from nine study sites. The information of sampling is provided in Table 1. Antibodies (IgG) to PUUV were found in 37 (13.55%) of the 273 voles included in the serological assays. Seroprevalence levels were highly variable (Table 1) and ranged between 0% (Sauville) and 43.3% (Hargnies). Among the 37 voles with anti-PUUV antibodies, only four had null PUUV viral load (*C*_*T*_>40 cycles, number of copies less than 10 per μg of vole RNA) and were considered as PUUV RNA negative in further statistical analyses. These individuals corresponded to three males (an immature and two old ones), and a gestant female. Note that three of these individuals were sampled in the 'crêtes pré-ardennaises'. In other PUUV-seropositive individuals, PUUV viral load ranged between 243 and 1 324 542 copies per μg of vole RNA.

**Table 1:** Description of the helminth diversity and PUUV seroprevalence per site of sampling.

Site of sampling	Landscape configuration	***N***_***v***_	***N***_***h***_**(*N***_***ces-larv***_***/N***_***ces-ad***_***/N***_***nem***_**)**	Dominant taxa	PUUV (%)
1-Hargnies	Forest	34	9 (1/2/6)	*Aonchoteca annulosa*	13 (43.33)
2-Woirie	Forest	37	7 (1/1/5)	*Heligmosomoides glareoli*	3 (8.82)
3-Renwez	Forest	38	7 (1/0/6)	*Heligmosomoides glareoli*	6 (16.67)
4-Cliron	Hedge	34	7 (2/1/4)	*Syphacia petrusewiczi*	3 (9.67)
5-Elan	Wood	27	5 (1/0/4)	*Heligmosomum mixtum*	2 (8.00)
6-Cassine	Wood	27	4 (1/1/2)	*Syphacia petrusewiczi*	6 (23.07)
7-Sauville	Hedge	31	8 (1/2/5)	*Syphacia petrusewiczi*	0 (0.00)
8-Croix-aux-bois	Wood	38	4 (1/0/3)	*Heligmosomoides glareoli*	3 (11.11)
9-Briquenay	Hedge	47	4 (2/0/2)	*Syphacia petrusewiczi*	1 (3.33)

*N*_*v*_, total number of voles trapped; *N*_*h*_, total number of helminth species observed per site; *N*_*ces-larv*, _number of cestode species in their larval stage; *N*_*ces-ad*_, number of cestode species in their adult stage; *N*_*nem*_, number of nematode species; PUUV, number of PUUV seropositive voles with corresponding prevalence in brackets.

The examination of the 313 digestive tracts allowed the detection of 12 helminth species, corresponding to nine genera. Seven were nematode species, among which six had direct cycles. Five were cestode species and they all had indirect cycles (Table 2). Bank voles experienced from none to five helminth species infection. The number of individuals of a given helminth species infecting a bank vole was highly variable (Table 2). Note that the numbers of *A. muris-sylvatici *and *T. crassiceps *worms were impossible to count.

**Table 2:** Description of the helminth species observed in *M. glareolus *trapped in the french Ardennes.

Species	Parasite group	Cycle (definitive or intermediate hosts)	Prevalence per site (range in %)	Number of helminths per vole (range, for non null values)
*Taenia taeniaeformis*	CES-LARV	I	[0-23.53]	[1-5]
*Taenia crassiceps*	CES-LARV	I	[0-2.94]	-
*Catenotaenia henttoneni*	CES-AD	I	[0-8.82]	[1-6]
*Hymenolepis (Arostrilepis s.l.) horrida*	CES-AD	I	[0-8.51]	[1]
*Paranoplocephala omphalodes*	CES-AD	I	[0-2.13]	[1]
*Mastophorus muris*	NEM	I	[0-17.65]	[1-12]
*Heligmosomoides glareoli*	NEM	D_i_	[2.63-44.44]	[1-17]
*Heligmosomum mixtum*	NEM	D_i_	[0-85.18]	[1-20]
*Trichuris arvicolae*	NEM	D_i_	[0-21.05]	[1-2]
*Syphacia petrusewiczi*	NEM	D_i_	[0-23.40]	[1-226]
*Aonchotheca annulosa*	NEM	D_i_	[0-8.82]	[1-70]
*Aonchotheca muris-sylvatici*	NEM	D_i_	[0-27.03]	-

NEM, nematodes; CES-LAR, cestodes infecting *M. glareolus *in their larval stage; CEST-AD, cestodes infecting *M. glareolus *in their adult stage; I, indirect cycle; D_i_, direct cycle. '-' indicates that helminth number could not be counted for the helminth species considered.

### PUUV infection risk factors

**Figure 2:**
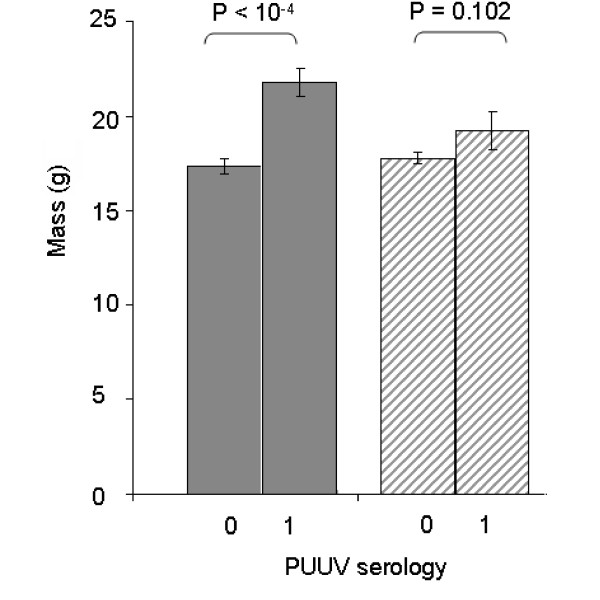
**Relationships between the mass (g) of bank voles and their seroprevalence with regard to PUUV (0: no anti-PUUV antibodies detected, 1: anti-PUUV antibodies detected) for each landscape configuration**. Grey bars represent data from the Northern sites (massif des Ardennes) and dashed bars correspond to the Southern sites (crêtes pré-ardennaises).

After the selection procedure, two equivalent models were obtained: PUUV ~ Site[Landscape] + Mass + Landscape*Mass (AIC = 286, Deviance ratio = 14.620, *p *< 10^-4^) or PUUV ~ Site[Landscape] + Sexual Maturity + Landscape* Sexual Maturity (AIC = 290, Deviance ratio = 7.401, *p *< 10^-4^). Body condition and sex were not significant. PUUV infection risk increased with mass or with sexual maturity, which both reflect the age of individual. This effect was mainly observed in the three northern sites (forests of the massif des Ardennes, see Figure 2). It was not significant when considering wooded areas and hedgerows of the southern part of the transect (crêtes pré-ardennaises), although a similar trend was observed.

### Helminth community structure and coinfection with PUUV

**Figure 3:**
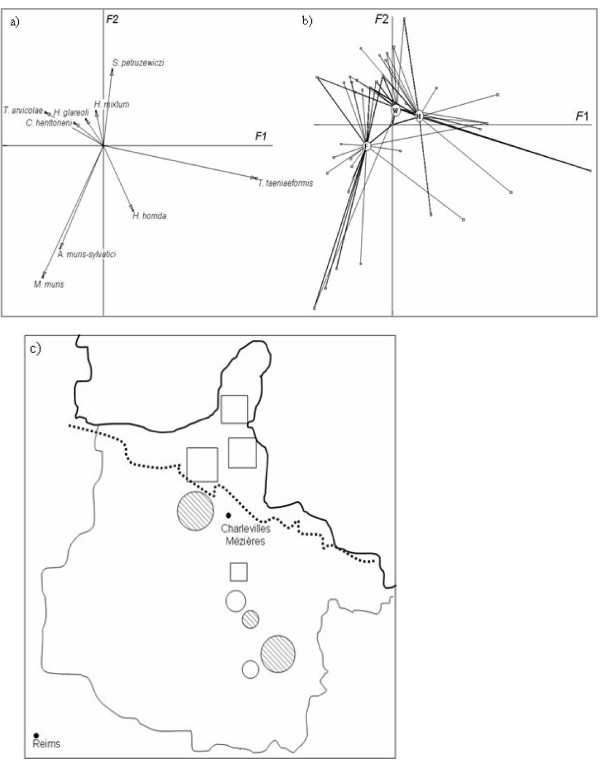
**Correspondence analysis of the helminth community structure**. a) Factorial plan (*F*1 × *F*2) showing the relationships between the helminth species. b) Factorial plan of the landscape according to its effect on the helminth community. The grey circles represent the gravity centres of the three landscapes considered, forest (F), wood (W) and hedge network (H). The lines show the variation within each site. c) Schematic representation of the site map based on helminth community characteristics. Sites represented with circles have above average *F*1 factorial values, whereas sites represented with squares have below-average *F*1 factorial values. Hedge networks are indicated with black dashed lines. Circle or square sizes are proportional to the distance of the value above or below the average value.

Three helminth species, namely *P. omphalodes*, *T. crassiceps *and *A. annulosa*, were too rare to be included in the multivariate analysis of the community structure. The first two factors (named hereafter *F*1 and *F*2) of the CA performed on the nine other helminth species described 30.08% of the variability. *T. arvicolae, M. muris and A. muris-sylvatici *had the highest correlations with the negative part of *F*1 (respective absolute contributions in 1/10000: 768, 752 and 442). *M. muris *and *A. muris-sylvatici *were also strongly correlated with the negative part of *F*2 (respective absolute contributions in 1/10000: 3733 and 2535). *T. taeniaeformis *was correlated with the positive values of *F*1 (absolute contributions in 1/10000: 7651) and *S. petrusewiczi *with the positive values of *F*2 (absolute contributions in 1/10000: 1392) (Figure 3a).

The factor 'Site of sampling' had a significant impact on both *F*1 and *F*2 axis values (Kruskal-Wallis, *p *< 10^-4^). This effect was mediated by the impact of 'Landscape configuration' (*F*1: Kruskal-Wallis, *p *< 10^-4^; *F*2: Kruskal-Wallis, *p *= 4 × 10^-4^, Figure 3b). Post-hoc Tukey Kramer tests showed that the helminth community observed in voles sampled in the Northern massif des Ardennes significantly differed from the one observed in voles sampled in the Southern part of the crêtes pré-Ardennaises, either in wooded or hedgerow areas. This result was confirmed when we projected the *F1 *or *F*2 values on the site map. Sites appeared divided into two areas, corresponding to the Northern massif des Ardennes and to the Southern crêtes pré-Ardennaises (Figure 3c). Most of the negative *F1 *values (squares) were located in the northern part of the area whereas the *F2 *positive values (circles) were observed in the southern part. By plotting the gravity centres of each landscape configuration on the *F*1x*F*2 factorial plan, it appeared that northern sites were characterized by the presence of *M. muris, A. muris-sylvatici *(they were not detected in Southern sites) and *T. arvicolae *whereas Southern sites experienced more infections associated with *T. taeniaeformis *and *S. petrusewiczi *(this latter species was not detected in Northern sites).

**Figure 4:**
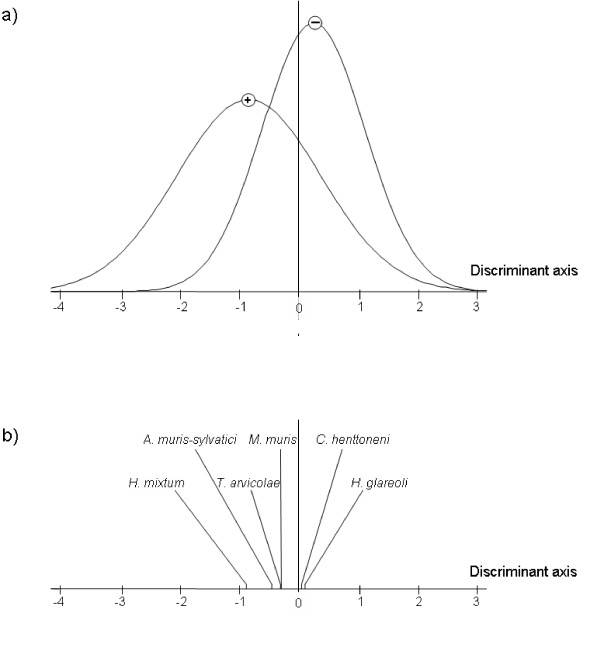
**Results of the discriminant analysis performed on the helminth community of PUUV-seronegative and PUUV-seropositive bank voles sampled in the northern sites of the transect**. a) Sample scores of the discriminant function for PUUV-seronegative and PUUV-seropositive bank voles. The symbols (-) and (+) represent the group averages of these two classes of individuals. b) Coefficient of the discriminant scores on this axis.

We therefore tested whether the helminth community varied between PUUV infected and non-infected bank voles. We analysed data independently for the Northern and the Southern parts of the transect. The discriminant analyses revealed significant differences when considering the northern area only (Massif des Ardennes, *p *= 0.005; Crêtes pré-ardennaises, *p *= 0.551, Figure 4a). The main discriminant species variable was the presence of *H. mixtum*, and in a lesser extent of *A. muris-sylvatici *(Figure 4b). Bank voles exhibiting anti-PUUV antibodies were more likely to be infected with these nematode species than bank voles with no anti-PUUV antibodies (*H. mixtum*: RR = 5.91, Fisher exact test: *p *= 0.002; *A. muris-sylvatici*: RR = 2.34, Fisher exact test, *p *= 0.125). We obtained similar results when comparing PUUV infected (with anti-PUUV antibodies and PUUV RNA) and non infected (without anti-PUUV antibodies or PUUV RNA) bank voles (*H. mixtum*: RR = 4.74, Fisher exact test: *p *= 0.007; *A. muris-sylvatici*: RR = 2.53, Fisher exact test, *p *= 0.102).

**Figure 5:**
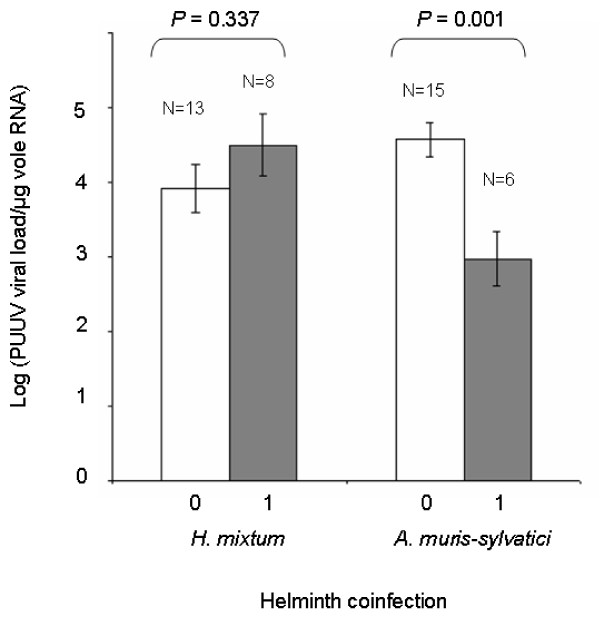
**Comparison of PUUV viral load in bank voles infected with *H. mixtum *or *A. muris-sylvatici *and in those not infected by these helminth species**. "0" indicates bank voles that are not infected with *H. mixtum *(resp. *A. muris-sylvatici*) and "1" indicates bank voles that are infected with at least 1 *H. mixtum *helminth (resp. *A. muris-sylvatici*). Only samples from the massif des Ardennes are considered. N indicates the sampling size for each category.

The viral load in infected individuals tended to be higher in voles coinfected with *H. mixtum *than in voles that did not carry any infection with this helminth species (*F*_*1,19 *_= 0.992, *p *= 0.331, Figure 5). Although the number of *H. mixtum *worms per vole had been counted, we could not analyse the relationship between PUUV viral load and *H. mixtum *burden. Indeed, among the eight voles that were coinfected by PUUV and *H. mixtum*, only one had more than one worm (this individual carried six *H. mixtum *worms), the seven other voles had only one *H. mixtum *worm. Surprisingly, voles coinfected with *A. muris-sylvatici *exhibited significantly lower viral load of PUUV than voles non-infected with this helminth species (*F*_*1,19 *_= 13.551, *p *= 0.001, Figure 5). As this negative relationship could be mediated by a delay between PUUV and *A. muris-sylvatici *infection, we analysed roughly the influence of vole age (reflected by vole mass) on these infections. We confirmed that voles coinfected with PUUV and *A. muris-sylvatici *were significantly heavier (thus probably older) than those infected with *A. muris-sylvatici *only, with PUUV only or non infected either with PUUV or *A. muris-sylvatici *(*F*_*3,96 *_= 7.279, *p *= 2 × 10^-4^).

## Discussion

Biomedical research has long explored the impact of coinfection on the outcome of human diseases [e.g. [27,28,44,45]]. Particular attention has been given to helminth-microparasite interactions, because host immune responses or immune regulation mediated by these pathogens generally have antagonistic effects [46].

So far, there are no studies on the interactions between helminths and hantaviruses even though helminth communities and PUUV distribution have been independently described for several natural populations of bank voles in the context of ecological, geographical and/or immunogenetic studies [e.g. [16,29,47-54]]. In a previous study, we combined macroparasites and PUUV infection data from bank vole populations sampled in the French Jura to analyse the relationships between immune gene variation and parasitism [52]. Unfortunately, the small number of PUUV-seropositive bank voles then prevented the possibility of searching for helminth-PUUV coinfection.

In this study, we combined serological and molecular methods to detect PUUV infection. Because PUUV infections are chronic in voles [55], the presence of antibodies is expected to be highly correlated with the presence of the virus. However during the breeding season, maternal antibodies might account for up to one third of the seropositive voles detected [56]. Moreover, previous studies in natural [57] or controlled [55] conditions have shown that the levels of shed hantavirus RNA could change a lot over time in excretion and blood samples. Although the highest rates of hantavirus shedding is generally observed during the first weeks after infection, viral RNA can be detected in blood for as much as 133 or 217 days post-infection [55,57]. Most of the PUUV antibody positive voles detected in this work were also PUUV RNA positive (33 out of 37). Among the four that had too low PUUV viral load to be considered RNA positive, one was an immature male. PUUV antibodies were likely to result from maternal transfer [e.g. [56,58]]. The three other voles were adults, and were probably not shedding PUUV at this time. We could however not investigate the reasons underlying these differences in PUUV viral load between PUUV antibody positive adult voles.

We used two appropriate methods to detect negative and positive interactions [43]. We reported significant positive associations between two helminth species (*H. mixtum *and *A. muris-sylvatici*) and PUUV infection in bank voles. Because helminths generally drive strong type 2 responses [59], which are antagonistic to type 1 responses involved in the immune defense against hantaviruses [review in [60]], we addressed the question of whether these helminth infections could influence vole susceptibility to PUUV.

First, we found that PUUV infection was more often observed in voles coinfected with *H. mixtum*, and that PUUV viral loads were slightly higher in voles coinfected with this nematode. These results can be interpreted with regard to the immune knowledge acquired from the close parasite *Nippostrongylus *(syn. *Heligmosomum*) *brasiliensis*, which is extensively used as a laboratory model to study Th2 immunity. In mice and rats, *N. brasiliensis *induces polarized Th2 responses characterized by elevation of IgE and Th2 cytokines such as IL-4, IL-5, and IL-13 [e.g. [61,62]]. This immune response might increase the susceptibility to PUUV. On another hand, Reece et al. [62] also reported that the baseline transcription levels of Th1 cytokines (IFN-γ, IL-12, and IL-6) are also elevated in *N. brasiliensis*-infected mice. This could explain that the Th2 response induced by *H. mixtum *is not strong enough to induce a dramatic increase of PUUV viral loads in coinfected voles. A similar observation had been made by Liesenfeld et al. [45] and Erb et al. [63] on a different biological system. They respectively showed that the densities of *Toxoplasma gondii *and *Mycobacterium bovis *in mice were only slightly affected by the presence of *N. brasiliensis. *Lastly, an added complexity in the interpretation of this coinfection is the possibility that it might be generated by correlated exposure, by parasite longevity and host age, or by differences in the genetic constitution of individual hosts. We can hypothesize that genetic factors of susceptibility might mediate the significant co-occurrence of PUUV and *H. mixtum *infection. Major histocompatibility complex (*Mhc*) class II genes could be relevant candidates as their polymorphism seems to influence the risk of PUUV or *H. mixtum *infection in bank voles [52,64,65]. Other candidate genes such as *Tnf-α*, which encodes for the Tumor Necrosis Factor alpha and strongly influence bank vole susceptibility to PUUV [66], should also be explored to better understand the potential influence of immunogenetics on the probability of helminth - PUUV coinfections.

Second, we found that PUUV viral loads were significantly decreased in voles coinfected with *A. muris-sylvatici*, although the risk of PUUV infection was slightly higher in voles coinfected with this nematode. Maturation status, which strongly influences the behaviour of voles and as such, has been shown to be a good determinant of parasite infection [29], could drive this slight and ambiguous pattern of co-occurrence observed between PUUV and *A. muris-sylvatici *infections [22]. Several studies have found that *Aoncotheca *species only occured in mature voles. These older individuals infected with *A. muris-sylvatici *were more likely to be infected with PUUV than younger ones as the risk of PUUV infection increases with age [e.g. [30,67,68]]. These PUUV infections could nevertheless have occurred earlier than those with *A. muris-sylvatici*, as suggested by the significant influence of vole mass (which reflects vole age) on the probability of single and co-infection. As bank voles secrete PUUV only during a limited time of the infection [55], the delay that is likely to exist between PUUV and *A. muris-sylvatici *infections could explain the low viral load observed in coinfected bank voles.

Besides, the lower loads of PUUV detected in voles coinfected with *A. muris-sylvatici *could also be the results of host immune response or immune regulators secreted by this nematode. A single study reported the immune consequences of *Aonchoteca *(syn = *Capillaria) *infection [69]. Although Kim et al. [69] showed an over-expression of genes encoding cytokines related to Th2 pathways, they also highlighted strong increases in the transcription levels of the Th1 cytokine IFN-γ. This cytokine is known to be crucial for restricting *Hantavirus *replication [review in [60]]. Indeed, IFN-γ is essential for inducing a variety of innate antiviral effector mechanisms such as natural killer (NK) cells or NKT cells [70,71]. The host is thus able to limit viral spread before the adaptive response is mounted. A suppressive effect of *A. muris-sylvatici *on PUUV viral replication could thus be mediated by the potential induction of IFN-γ production following *A. muris-sylvatici *infection.

Our study also stressed the main importance of considering landscape configuration when analysing patterns of coinfection, especially in the case of helminths and PUUV.

First, we showed that the helminth community structure of bank voles was strongly affected by landscape. Main differences were observed between the Northern massif des Ardennes and the Southern crêtes pré-ardennaises. *S. petrusewiczi *was for example never recorded in the Northern sites while *H. horrida*, *M. muris *and *T. arvicolae *were extremely rare in the Southern sites. Helminths are known to interact with the external environment. Climatic factors or soil composition are examples of conditions that may affect the development of their free-living stages or the survival of their transmission stages outside their hosts [e.g. [72-75]]. The distinction between the Northern massif des Ardennes and the Southern crêtes pré-ardennaises relies on geological and climatic differences that could in turn explain geographical variations in the helminth community structure. Indeed, the Northern massif is characterized by primary soils (shist, slate), cold winters and higher precipitations whereas the crêtes pré-ardennaises are composed of secondary soils (clay) and experience less severe winter and rainfall. Besides, we found no differences between the helminth communities observed in wooded areas and hedgerows from the Southern area. This was surprising because population genetic analyses have revealed that bank vole populations from hedgerows experienced strong genetic drift, leading to strong genetic differentiation among them and between populations from hedgerows and wooded areas [76]. It is possible that both bank vole dispersal from wooded areas to hedgerows, as well as the existence of survival stages in the external environment, might counterbalance the impact of drift on the helminth community structure of hedgerows.

This spatial differentiation of helminth communities observed between the northern massif and the southern cretes could lead to false associations mediated by the distribution of particular species. The same observation holds for PUUV as we showed that its distribution also exhibited strong disparities between sites. Several studies have stressed the influence of environmental factors, including winter temperature and soil moisture, on PUUV prevalence in bank vole populations [15,19]. Deeper insights into local factors mediating differences in quality of forest patches could provide a better understanding of the spatial variations of PUUV prevalence mediated by variations in bank vole abundance or dynamics [31,77]. Particular attention could especially be given to the differences in proportions of functional groups (e.g. mature *vs *immature voles) mediated by environmental and landscape variations, as PUUV and helminth species structures strongly depend on these proportions.

Finally, landscape configuration and environmental conditions might enhance or deplete the possibility for immune-mediated coinfection to occur. High population densities, and low availability of resources, might constitute stressful environmental factors that can in turn lead to trade-offs between fitness components [78], and even between immune pathways [79,80]. Immune responses that are energetically costly (e.g. systemic inflammatory response) are expected to be depleted at the expense of less costly ones (e.g. antibody-mediated immunity). Therefore, spatio-temporal variations in environmental factors influencing the costs and benefits of resistance to PUUV of gastro-intestinal helminths could promote geographic differences in the occurrence of coinfections. This process might participate in explaining why PUUV - *H. mixtum *coinfection are only detected in the Northern massif des Ardennes despite the presence of *H. mixtum *over the region sampled. The Southern crêtes pré-ardennaises might experience less stressful climatic conditions that do not lead to strong trade-offs between immune responses. Temporal surveys of helminths and PUUV in these two geographic areas and in other part of Europe could help confirming this hypothesis. Such longitudinal studies, including different sampling seasons, could also bring insight into the influence of population age structure in the helminth-PUUV interactions described here.

## Conclusions

To our knowledge, this is the first study that analyses hantavirus - helminth coinfection in natural populations of reservoirs. Our research stressed the influence of the environment in enhancing or depleting the occurrence of these coinfections. PUUV and parasite species distributions, which strongly depend on soil and climatic factors, and immune trade-offs mediated by stressful environmental conditions may affect the incidence and our capacities to detect coinfections of biological significance. Longitudinal studies are now required to follow the same marked bank voles through times and to disentangle the host, pathogen and environmental factors underlying the PUUV-helminth associations described in this study.

## Competing interests

The authors declare that they have no competing interests.

## Authors' contributions

EG, JFC and NC conceived the study, participated in its design and carried out its coordination. ARS prepared samples, collected and analysed helminth data (identification and and counting). AX, YC, JFC, EG, ARS and MLP participated in the field work. PC participated in analyzing the data. TS and HH analysed PUUV viral load data. LV and HH analysed PUUV serological data. NC drafted the manuscript. All authors read, criticized and approved the final manuscript.
